# The Prognostic Impact of Quitting Smoking at or around Diagnosis on the Survival of Patients with Gastrointestinal Cancers: A Systematic Literature Review

**DOI:** 10.3390/cancers14163857

**Published:** 2022-08-09

**Authors:** Saverio Caini, Marco Del Riccio, Virginia Vettori, Sara Raimondi, Melania Assedi, Silvano Vignati, Guglielmo Bonaccorsi, Maria Sofia Cattaruzza, Federica Bellerba, Giulia Vagnoni, Giacomo Duroni, Sara Gandini

**Affiliations:** 1 Cancer Risk Factors and Lifestyle Epidemiology Unit, Institute for Cancer Research, Prevention and Clinical Network (ISPRO), 50139 Florence, Italy; 2 Postgraduate School in Hygiene and Preventive Medicine, University of Florence, 50134 Florence, Italy; 3 Department of Health Sciences, University of Florence, 50134 Florence, Italy; 4 Department of Experimental Oncology, European Institute of Oncology (IEO), IRCCS, 20141 Milan, Italy; 5 Department of Public Health and Infectious Diseases, Sapienza University of Rome, 00185 Rome, Italy

**Keywords:** gastrointestinal cancer, colorectal cancer, gastric cancer, liver cancer, smoking cessation, survival, systematic review, meta-analysis

## Abstract

**Simple Summary:**

Smokers are at high risk of cancer of the gastrointestinal system, and many patients with newly diagnosed cancer of the oesophagus, stomach, colon-rectum, and liver are active smokers at diagnosis. In this review, we focused on whether stopping smoking shortly before diagnosis or afterwards (e.g., during treatment) may improve the chance of survival for these patients. We reviewed the scientific literature up to April 2022 and found only seven articles focusing on this topic. While very limited in number, these studies provided suggestive evidence in favour of a beneficial effect of smoking cessation for these patients. Smokers with newly diagnosed cancer of the gastrointestinal system should be encouraged to stop smoking and provided all the necessary support to achieve this goal.

**Abstract:**

Cigarette smoking is a strong risk factor for the occurrence of gastrointestinal cancers, and a substantial proportion of newly diagnosed patients is made up of active smokers, yet the impact of smoking cessation at or around diagnosis on the clinical course of these cancers (whose prognosis is often unfavourable) has never been summarized to date. We reviewed studies published until 30 April 2022 that investigated whether smoking cessation at or around diagnosis favourably affects the clinical course of gastrointestinal cancers patients. Six studies were included for colorectal cancer patients, which provided limited yet suggestive evidence that quitters may have longer disease-specific survival compared to continued smokers. Only one study each focused on patients with gastric or HBV-positive liver cancer (both reporting a survival advantage for quitters vs. continued smokers), while we found no eligible studies for patients with cancer at other sites within the digestive system. More research is urgently needed to expand the evidence on the topic, given the potentially major clinical implications for these patients. Moreover, health professionals should provide the necessary smoking cessation support to any smoker who is undergoing diagnostic work-up or treatment for gastrointestinal cancer.

## 1. Introduction

Gastrointestinal cancers (which encompass cancers of the gastrointestinal tract as well as of accessory organs) impose a heavy burden of disease on human populations worldwide. According to the GLOBOCAN 2020 estimates, cancers of the colorectum, liver, stomach, oesophagus, and pancreas rank among the top seven cancers in terms of both incidence (when excluding nonmelanoma skin cancers) and mortality rates globally [1]. In 2020, those five cancer types were estimated to have caused around 3.4 million deaths, which corresponds to more than one third of all cancer-related deaths globally [1]. The global burden of disease of oesophageal and gastric cancers has generally been decreasing over the past few decades, while greater geographical diversity exists for cancers of the colorectum, liver, and pancreas, whose incidence and mortality rates have been on the rise in some world regions and stable or even decreasing elsewhere [2].

Established risk factors exist for all gastrointestinal cancer types, several of which are potentially modifiable and therefore amenable to primary prevention. While some exposures are linked with cancer at specific sites within the digestive system (e.g., *Helycobacter pylori* for gastric cancer and HBV and HCV for liver cancer), others are common to more cancer sites, and, in particular, cigarette smoking is responsible for a substantial fraction of most gastrointestinal cancers globally. In detail, the percentage of disability-adjusted life years (DALYs) lost to gastrointestinal cancers in 2017 that were caused by cigarette smoking is estimated to be 39% for oesophageal cancer [3], 17.1% for gastric cancer [4], 13.3% for colorectal cancer [5], and 21.1% for pancreatic cancer [6]. Cigarette smoking causes a non-negligible proportion of hepatocellular cancer as well, although the figures vary considerably across regions due to the highly varying prevalence of other risk factors such as alcohol intake, HBV and HCV infection, exposure to aflatoxins, and others [7,8,9].

Tobacco smoking remains ubiquitous worldwide and, given its association to gastrointestinal cancer risk, it is very likely that a substantial proportion of these patients are still actively smoking at the moment of diagnosis. It is, therefore, of considerable interest to investigate whether these patients can obtain a prognostic benefit by stopping smoking at diagnosis, shortly before it (e.g., during diagnostic workup), or afterwards (e.g., during treatment). Besides initiating and promoting cancer development at multiple sites, continued smoking is thought to also hold prognostic significance through various mechanisms, e.g., by facilitating the dissemination of cancer cells and the formation of distant metastases, or by conferring resistance to therapy and increasing its side effects. We recently reviewed and meta-analysed the evidence available for lung cancer, and showed that smokers who quit at or around diagnosis may substantially extend their survival [10]. Whether this also holds for subjects with gastrointestinal cancers is currently unclear, and no attempt has ever been made, to our knowledge, to systematically review the available evidence on this subject. Aiming to fill this knowledge gap, we conducted a systematic review and meta-analysis on the association between smoking cessation at/around diagnosis and the prognosis of gastrointestinal cancer patients.

## 2. Materials and Methods

The protocol of the present systematic review and meta-analysis can be accessed in PROSPERO (registration number CRD42021245560) [11]. The PRISMA (Preferred Reporting Items for Systematic Reviews and Meta-analysis) 2020 statement was followed while planning and conducting this systematic review and reporting its results [12].

### 2.1. Search Strategy

The literature search was conducted in MEDLINE and EMBASE on 30 April 2022, using the following string: (smok*) AND (cease OR cessation OR quit* OR stop*) AND (cancer OR carcinoma OR tumo(u)r OR malignancy) AND (survival OR prognos* OR outcome OR mortality). No restrictions were applied (e.g., by publication date or geographic region), and all papers were considered for which an English abstract was provided.

### 2.2. Study Selection

Upon removing duplicate items, two independent researchers (MDR and VV) screened the titles and abstracts of all retrieved papers and discarded those that focused on different subjects. Eligibility for inclusion was decided upon reading of the full text: in case of disagreement, a consensus choice was made based on advice from a third senior researcher (SC or SG). Further eligible articles were searched for by backward citation chaining and by perusing the reference list of previously published reviews and meta-analyses, if any. Eligible studies were those that reported (or allowed to calculate, see below) a hazard ratio (HR) and corresponding 95% confidence intervals (CI)—or another measure of statistical uncertainty such as standard errors, variance, or exact *p*-values—for the association between smoking cessation at diagnosis, shortly (up to 1 year) before it, or afterwards (e.g., during treatment), and the survival of patients diagnosed with cancer of the gastrointestinal tract (codes for oncology C15-C26 in the ICD-10 classification [13]), namely cancer at any of the oesophagus, stomach, small intestine, colon, rectosigmoid junction, rectum, anus and anal canal, liver and intrahepatic bile ducts, gallbladder and other extrahepatic bile ducts, and pancreas. Overall survival (OS) and disease-specific survival (DSS) were considered, alongside progression-, recurrence-, and disease-free survival. Studies that failed to report the exact timing of smoking cessation were not considered for inclusion in the present review, as well as papers that did not report original data (e.g., editorials, commentaries, and letters without data).

### 2.3. Data Extraction

The following information was extracted from all eligible studies and inputted into an internally piloted spreadsheet: country and year of conduction of the study; study design; number of cancer patients who were smokers at diagnosis and their demographics (age and sex); number of quitters and continued smokers, alongside the exact definition of both groups; distribution of the main tumour characteristics (e.g., site, histological subtype, stage and grade) and the number of patients who underwent any different treatment type; length of follow-up (median and maximum); details on the statistical methods used to calculate the association between smoking cessation and patients’ survival and what variables were used for adjustment, if any. The HR and 95% CI for the association between smoking cessation and patients’ survival were inverted whenever necessary in order to make the subset of patients coded as “continued smokers” as the category of reference, and then transformed into log(HR) and corresponding variance according to Greenland’s formula [14]. If no HR was reported by the study authors but the survival curves for quitters and continued smokers were available in the text, an unadjusted HR and corresponding 95% CI was worked out by applying Parmar’s method [15].

### 2.4. Statistical Analysis and Study Quality Assessment

We had planned to merge study-specific HRs into summary HRs by means of random effects models with maximum likelihood estimation [16]; quantify heterogeneity of HRs across studies by using the I^2^ statistics [17]; and run meta-regression, subgroup analysis, and leave-one-out sensitivity analysis to identify sources of the observed heterogeneity (in case I^2^ exceeded 50%) [18], alongside Egger’s and Begg’s tests to check for publication bias [19,20]. However, the eligible studies were very limited in number, so it was not possible to conduct a meta-analysis, and study-specific results were instead reported in tables and commented in the text without making any attempt to obtain summary measures of association. This was partly caused by the studies differing in how the results of interest were reported. In some studies, quitters and continued smokers were directly compared in terms of their survival, while in other studies (which were eligible for inclusion in the review, but could not be used for meta-analysis purposes) a third group of patients (never smokers, or all those who were not active smokers at diagnosis, including both never and long former smokers) served as a reference to which quitters and continued smokers were separately compared. Finally, the study quality and susceptibility to bias was evaluated by using the Quality in Prognosis Studies (QUIPS) tool [21].

## 3. Results

**Figure 1:**
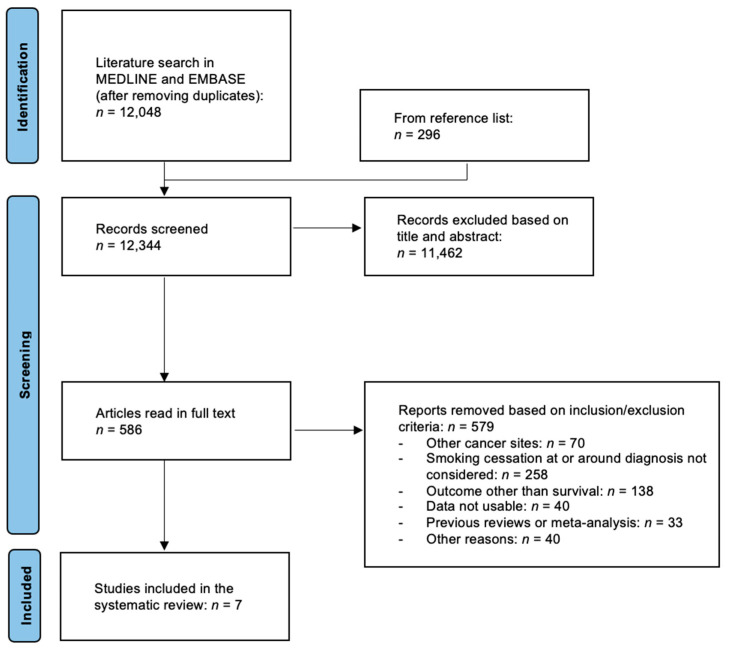
Flow-chart of the literature search and articles selection for the systematic review and meta-analysis on the effect of quitting smoking at or around diagnosis on the survival of patients with cancer of the gastrointestinal tract.

The literature search returned 12,048 non-duplicate articles, and an additional 296 were found in reference lists (Figure 1). A total of 11,462 articles were excluded based in their title or abstract, leaving 586 articles that were read in full text. Of these, 579 were not eligible because they focused on other cancer sites (*n* = 70), did not consider smoking cessation at/around diagnosis (*n* = 258), failed to report on the association between smoking cessation and cancer patients’ survival (*n* = 138), and various other reasons (*n* = 113). Among the studies that were found not to be eligible, that by Yang and colleagues could not be included because the timing of pre- and post-diagnosis smoking assessment (2.3 years before diagnosis and 1.4 years after diagnosis on average, respectively) did not strictly match the criteria that we had set for eligibility [22]. Still, the findings were of interest for the present review, as quitters were found to have a survival advantage compared to continued smokers: the HR comparing the DSS of either group to that of never/former smokers was 1.85 (95% CI 1.02–3.35) for the former and 2.20 (95% CI 1.29–3.76) for the latter.

Seven articles published between 2011 and 2020 were finally found eligible for the present review (Table 1): of these, five included subjects diagnosed with colorectal cancers [23,24,25,26,27], the study by Tao et al. encompassed patients with cancers of the stomach and of the colorectum (as well as with cancers at extra-gastrointestinal sites, not considered here) [28], and the study by Zhang et al. focused on subjects with HBV-positive liver cancer [29]. No eligible studies were found that reported on patients with malignancies of the oesophagus, small intestine, extrahepatic bile ducts, or the pancreas. The percentage of patients that were active smokers at diagnosis (or had quit less than 1 year before it) ranged from 46% for colorectal cancer patients in the study by Tao et al. to 13.8% in the study by Walter et al. (Table 1). The distribution of tumours across stages at diagnosis, the proportion of patients that underwent the different available treatments, and the duration of patient’s follow-up were very diverse across studies (for some studies, this information was not made available to readers) (Table 1). In four studies, the category of “quitters” included patients who had stopped smoking within 1 year after cancer diagnosis [23,27,28,29], while in the remaining three studies, patients who had stopped smoking by less than 1 year before diagnosis were also included in the study as “quitters” [24,25,26] (Table 2). Of note, in none of the studies was an objective method (e.g., cotinine concentration in urine or saliva, or exhaled carbon monoxide) used for the determination of patients’ smoking status, which relied therefore entirely on self-reporting: this relative lack of validity in the description of the prognostic factor was the most important limitation in the included studies, alongside with the frequent lack of information on the proportion of patients that was lost to follow-up (as well as patients’ and tumour characteristics) and the inability to report an adjusted HR directly comparing the survival of quitters vs. continued smokers in three studies (Supplementary File S1).

We found no strong evidence overall that quitting at or around diagnosis is effective in extending the OS of colorectal cancer patients (Table 3). The hazard for all-cause mortality was significantly reduced among quitters vs. continued smokers only in the study of Tao et al. (HR 0.29, 95% CI 0.14–0.59), while results were largely conflicting in the remaining studies. Suggestively, a survival advantage emerged among quitters with stage IV colorectal cancer patients (unlike among stage I-III patients, where no effect of smoking cessation was observed) in the study by Walter et al., which was, however, the only study to report the results of analyses stratified by tumour stage. Concerning the DSS of colorectal cancer patients, all three included studies reported a survival advantage for quitters compared to continued smokers (Table 3). In the study by Walter et al., the HR for comparison to the group of never/former smokers was 0.87 for quitters (95% CI 0.60–1.25) and 1.10 for continued smokers (95% 0.83–1.45). Among female colorectal cancer patients in the study by Warren et al., quitters had a non-significantly 15% lower hazard of disease-specific death compared to continued smokers (HR 0.85, 95% CI 0.25–2.94; results were not available for males). Moreover, quitters were also found to have a somewhat improved DSS compared to continued smokers in the study by Phipps and colleagues: compared to never smokers, the HR was 1.32 (95% CI 1.00–1.74) for the former, and 1.47 (95% CI 1.07–2.03) for the latter (Table 3). The association between at/around diagnosis smoking cessation and colorectal cancer patients’ recurrence-free and disease-free survival was investigated only in the study by Walter et al. 2015: for both endpoints, the HR for comparison to never/former smokers was lower among the quitters (1.00, 95% 0.73–1.36 and 1.04, 95% 0.78–1.39, respectively) than among the continued smokers (1.18, 95% CI 0.93–1.51 and 1.18, 95% CI 0.95–1.48). Smokers diagnosed with gastric cancer who quit at or around diagnosis had a non-significant 26% reduction in the hazard of death (95% CI 0.37–1.43) compared to those who continued smoking in the study by Tao et al. (Table 3). Finally, cessation in the first year after diagnosis was found to be associated with a more-than-halved risk of recurrence among smokers with HBV-positive liver cancer in the study by Zhang et al.

## 4. Discussion

We systematically reviewed the published studies that examined the association between smoking cessation at or around diagnosis and the prognosis of patients with gastrointestinal cancer. Among the articles that were included, six focused on colorectal cancer, one each on gastric and HBV-positive liver cancer, and none on cancer at other sites within the digestive system (i.e., the oesophagus, small intestine, gallbladder and extrahepatic bile ducts, and anus and anal canal). Due to the small number of available studies and the large heterogeneity in the way the study findings were presented, we were not able to apply meta-analysis methods to work out summary measures of association as originally planned. Nonetheless, the results of the few available studies did provide some indication, albeit still very preliminary and in need of confirmation, that cessation during diagnostic workup or before and during treatment may indeed lead to an appreciable clinical benefit for smokers diagnosed with gastrointestinal cancer.

Multiple mechanisms have been hypothesized to explain the observed association of cigarette smoking (and continuation or cessation thereof after diagnosis) with the survival expectations of gastrointestinal cancer patients. Continued exposure to nicotine and other tobacco smoke components can lead to sustained colon tumour growth (through both increased proliferation and decreased apoptosis), enhanced angiogenesis, increased invasiveness, impaired responsiveness to chemotherapy, including cetuximab-based treatment, silencing of multiple regulatory genes via epigenetic mutations, and emergence of mutant clones able to escape systemic treatments [30,31,32,33,34,35]. Finally, continued smoking may unfavourably alter host characteristics, e.g., by worsening underlying comorbidities and impairing immunity, further undermining a patient’s chance of recovering from the disease.

This is the first systematic review of published articles that studies whether smoking cessation around diagnosis impacts the prognosis of gastrointestinal cancers, and although the small number of available studies prevented a meta-analysis to be conducted and undermined the strength of the conclusions that could be drawn, it still has the merit of having identified possible causal links and knowledge gaps, thus suggesting future lines of research that may eventually produce results with vast clinical impact as well as immediate translability. Besides the quantity of studies, however, it is essential that the quality of the evidence is also enhanced. In this regard, a study adequately designed to answer this scientific question should at least apply a reliable and reproducible method of verifying effective smoking cessation, an efficient method of removing statistical confounding (as the conduction of an RCT would not be ethical in this setting), and the systematic reporting of results stratified for major prognostic factors such as tumour stage, molecular phenotypes (e.g., for colorectal cancer, e.g., microsatellite instability and BRAF and KRAS status), HBV and HCV status (for hepatocellular cancer), any treatment undergone by the patient, and others. In addition, the present line of research could be further expanded by conducting (or systematically reviewing and meta-analysing) studies focusing on the effect of smoking cessation on other patients’ endpoints in addition to survival (e.g., quality of life), or aiming at comparing and quantifying the effectiveness of alternative smoking cessation programs directed to cancer survivors. With reference to the latter point, it is worth highlighting that the evidence on the effectiveness of alternative smoking cessation programs (e.g., in-person counselling, quit lines, etc.) specifically directed to cancer survivors is still very limited (particularly, very few RCTs have been conducted to date) [36,37], thus more research in this area would be greatly and urgently needed.

## 5. Conclusions

In conclusion, we find limited yet suggestive evidence that managing to quit smoking at or around diagnosis may convey some prognostic benefit to smokers diagnosed with colorectal, gastric, or HBV-positive liver cancer. Although very limited, the findings of the existing papers should not be overlooked and make a strong argument in favour of conducting additional studies on the topic. By and large, in fact, any chance to obtain a gain in survival for cancer patients should not be dismissed, and this is all the more important in this setting when considering that the prognosis of gastrointestinal cancers is generally unfavourable, and actually frankly dismal for cancer of the pancreas, oesophagus, and liver. Moreover, stopping smoking has the potential to bring health benefits (besides the potential impact on the clinical course of gastrointestinal cancers) that are impossible to overestimate and include a sharp decrease in the risk of developing subsequent smoking-related illnesses (e.g., second primary tumours) and substantial improvements in the ability to cope with underlying diseases and in the overall quality of life [38,39]. Moreover, substantial improvements in cancer survivors’ quality of life might also be brought about by smoking cessation. While more evidence is urgently needed on the subject of the present review, health professionals must in no way hesitate in advising to quit smoking (and providing all the required support) to any smoker they encounter during their professional activity, regardless of the severity of the illness he or she may suffer from, as recommended by the World Health Organization’s MPOWER package [40]. To this regard, it is important to acknowledge that one of the main barriers encountered by health professionals in advising patients to quit smoking is insufficient training on tobacco dependence and smoking cessation [41]. Thus, the need to disseminate evidence-based guidelines and implement training programs to enhance the performance of health professionals in offering tobacco dependence treatment to their smoking patients should be emphasized [42].

## Figures and Tables

**Table 1:** Main characteristics of the studies included in the systematic review on the prognostic effect of quitting smoking at or around diagnosis on the survival of patients with cancer of the gastrointestinal tract.

Author, Year	Country	Sex (% Men)	Age (Years)	Cancer Site	Smoking Status			Years of Diagnosis	Tumour Stage	Treatments	Follow-Up Duration (Years)
					Non-Smokers at Diagnosis ^(a)^	Continued Smokers	Quitters				
Jang, 2020 ^(b)^	South Korea	100.0%	<60: 38.6%, ≥60: 61.4%	colon (100%)	15,564 (71.4%)	2420 (11.1%)	3808 (17.5%)	2002–2016	NA	surgery alone (67.7%), surgery with RT or CHT (28.6%), RT or CHT alone (3.7%)	median 6.3
				rectum (100%)	10,415 (68.1%)	1756 (11.5%)	3116 (20.4%)				
Japuntich, 2019	USA	45.1%	<60: 35.5%, ≥60: 64.5%	colon-rectum (100%)	2634 (84.7%)	289 (9.3%)	187 (6.0%)	2003–2005	I, II (47.7%), III (39.8%), IV (12.5%)	surgery (57.9%), CHT (33.0%), RT (9.1%)	max 7.0
Walter, 2015	Germany	59.3%	median 69 (range 30–96)	colon (59.2%), rectum (40.8%)	2690 (86.2%)	276 (8.9%)	153 (4.9%)	2003–2010	I (22.2%), II (30.8%), III (32.7%), IV (14.3%)	surgery (100%), other treatments (NA)	median 4.9
Zhang, 2014	China	83.8%	mean 48.9	liver HBV+ (100%)	193 (63.9%)	22 (7.3%)	87 (28.8%)	2008–2011	NA	surgery + HBV treatment (100%)	median 2.2
Tao, 2013 ^(c)^	China	NA	NA	stomach (100%)	227 (62.7%)	135 (% in either category NA)		1986–2010	NA	surgery, CHT, RT (% NA)	mean 5.3
		NA	NA	colon-rectum (100%)	134 (54.0%)	114 (% in either category NA)		1986–2010	NA	surgery, CHT, RT (% NA)	
Warren, 2013	USA	56.6%	mean 60.2	colon-rectum (100%)	291 (81.1%)	47 (13.1%)	21 (5.8%)	1982–1998	local (27.3%), regional (36.2%), distant (36.5%)	NA	min 12.0, max 27.7
Phipps, 2011	USA	53.7%	range 18–74	colon (64.3%), rectum (34.4%), NA (1.3%)	1.851 (81.9%)	152 (6.7%)	258 (11.4%)	1998–2007	I (32.1%), II (15.2%), III (12.5%), IV (20.1%), NA (20.1%)	NA	max 12.0

^(a)^ This category includes never smokers and long former smokers. ^(b)^ Results were reported only separately for patients with colon and rectal cancer. ^(c)^ Data on patients’ demographics (sex and age), smoking habits, and treatment was available only for the entire study sample, which included patients with cancer at various sites. NA: not available. HBV: hepatitis B virus. CHT: chemotherapy. RT: radiotherapy.

**Table 2:** Definition of quitters and continued smokers in the studies included in the systematic review on the prognostic effect of quitting smoking at or around diagnosis on the survival of patients with cancer of the gastrointestinal tract.

Author, Year	Quitters	Continued Smokers
Jang, 2020	Stopped smoking within 1 year after diagnosis.	Continued to smoke in the first year after diagnosis.
Japuntich, 2019	Quit less than 1 year before diagnosis.	Active smokers at diagnosis.
Walter, 2015	Quit less than 1 year before diagnosis.	Active smokers at diagnosis.
Zhang, 2014	Quit smoking within 1 year of diagnosis.	Continued smokers after surgery, for at least 1 year or until death.
Tao, 2013	Never smoked cigarettes after diagnosis.	Continued to smoke until death or the latest follow-up interview.
Warren, 2013	Quit less than one year before diagnosis.	Active smokers at diagnosis.
Phipps, 2011	Quit smoking at the post-diagnosis interview (median 6.9 months after diagnosis).	Continued to smoke at the post-diagnosis interview.

**Table 3:** Hazard ratio (HR), 95% confidence intervals (CI), and details of the statistical analysis for the association between at/around diagnosis smoking status (cessation/continuation) and survival of patients with cancer of the gastrointestinal tract.

Author, Year	Smoking Status	HR	95% CI	Variables Used for Statistical Adjustment
**Colorectal Cancer, Overall Survival**				
Jang, 2020	continued smokers	1.00 (ref)		age, comorbidities, alcohol intake, BMI, physical activity levels
	quit smoking (colon)	1.06	0.90–1.25	
	quit smoking (rectal)	1.10	0.92–1.32	
Japuntich, 2019	continued smokers	1.00 (ref)		age, sex, tumour stage, comorbidities, alcohol intake, BMI, other
	quit smoking	0.87	0.64–1.18	
Walter, 2015	never + former smokers	1.00 (ref)		age, sex, tumour stage, comorbidities, alcohol intake, BMI, other
	continued smokers	1.10	0.86–1.41	
	quit smoking	0.97	0.70–1.33	
Tao, 2013 ^(a)^	continued smokers	1.00 (ref)		age, pack-years, treatment received, other
	quit smoking	0.29	0.14–0.59	
Warren, 2013 ^(a) (b)^	continued smokers	1.00 (ref)		age, tumour stage, pack-years, BMI, other
	quit smoking	1.19	0.44–3.33	
Phipps, 2011	never smokers	1.00 (ref)		age, sex, other
	continued smokers	1.50	1.14–1.97	
	quit smoking	1.52	1.21–1.90	
**Colorectal cancer, disease-specific survival**				
Walter, 2015	never + former smokers	1.00 (ref)		age, sex, tumour stage, comorbidities, alcohol intake, BMI, other
	continued smokers	1.10	0.83–1.45	
	quit smoking	0.87	0.60–1.25	
Warren, 2013 ^(a) (b)^	continued smokers	1.00 (ref)		age, tumour stage, pack-years, BMI, other
	quit smoking	0.85	0.25–2.94	
Phipps, 2011	never smokers	1.00 (ref)		age, sex, other
	continued smokers	1.47	1.07–2.03	
	quit smoking	1.32	1.00–1.74	
**Colorectal cancer, recurrence-free survival**				
Walter, 2015	never + former smokers	1.00 (ref)		age, sex, tumour stage, comorbidities, alcohol intake, BMI, other
	continued smokers	1.18	0.93–1.51	
	quit smoking	1.00	0.73–1.36	
**Colorectal cancer, disease-free survival**				
Walter, 2015	never + former smokers	1.00 (ref)		age, sex, tumour stage, comorbidities, alcohol intake, BMI, other
	continued smokers	1.18	0.95–1.48	
	quit smoking	1.04	0.78–1.39	
**Stomach cancer, overall survival**				
Tao, 2013 ^(a)^	continued smokers	1.00 (ref)		age, pack-years, treatment received, other
	quit smoking	0.74	0.37–1.43	
**Liver cancer (HBV-positive patients), recurrence-free survival**				
Zhang, 2014	continued smokers	1.00 (ref)		none (extracted from KM curve)
	quit smoking	0.41	0.25–0.65	

The HR and corresponding 95% CI were inverted in order to make continued smokers the reference category. ^(b)^ Results were reported only for female patients in the paper. HR: hazard ratio. CI: confidence intervals. BMI: body mass index. KM: Kaplan–Meier.

## Supplementary Materials

The following supporting information can be downloaded at: https://www.mdpi.com/article/10.3390/cancers14163857/s1, Supplementary File S1 (spreadsheet): Quality assessment of the included studies using the QUIPS (Quality in Prognostic Studies) tool.
